# Adsorption Characteristics of an AlGaN/GaN Heterojunction on Potassium Ions

**DOI:** 10.3390/molecules30132669

**Published:** 2025-06-20

**Authors:** Yan Dong, Mengmeng Li, Yanli Liu, Jianming Lei, Haineng Bai, Yanmei Sun, Dunjun Chen, Dongjie Zhu, Rigao Wang, Yi Sun

**Affiliations:** 1 College of Electrical Engineering, Zhejiang University of Water Resources and Electric Power, Hangzhou 310018, China; 2 Zhejiang-Belarus Joint Laboratory of Intelligent Equipment and System for Water Conservancy and Hydropower Safety Monitoring, Hangzhou 310018, China; 3 School of Electronic Engineering, Heilongjiang University, Harbin 150080, China; 4 School of Information and Electronic Engineering, Shandong Technology and Business University, Yantai 264005, China; 5 School of Electrical Engineering, Nanjing Institute of Industry Technology, Nanjing 210023, China; 6 School of Physics and Telecommunication Engineering, Zhoukou Normal University, Zhoukou 466001, China; 7 School of Electronic Science and Engineering, Nanjing University, Nanjing 210093, China

**Keywords:** AlGaN surface, potassium ion, AlGaN/GaN HEMT

## Abstract

Slight changes in potassium levels can affect health. Therefore, rapid, reliable, and quantitative determination of potassium ion content is important for medical diagnosis. AlGaN, as a semiconductor material with good biocompatibility, has many advantages in the development of new potassium ion sensors. Understanding the adsorption behavior of a specific ion on the AlGaN surface and the eventual effect on AlGaN/GaN’s heterostructure interface is the key to obtaining high-performance nitride sensors. In this paper, we calculated the changes in the density of states and energy bands of the material after AlGaN adsorbed potassium ions through first-principles simulation. Combined with two-dimensional device simulation software, the changes in device performance caused by the changes in material properties are presented. The simulation results show that the adsorption of a single potassium ion can cause a current change in the order of milliamperes, providing a theoretical reference for the subsequent development of high-sensitivity potassium ion sensors.

## 1. Introduction

AlGaN/GaN heterojunctions can produce high concentrations of two-dimensional electron gas through polarization alone without doping and can sense any small changes in the vertical heterogeneous interface, and closer to the surface, they are more sensitive to surface charge, so they could also be advantageous in the field of sensors [1,2]. And GaN materials are non-toxic and have better biocompatibility and excellent material properties, which makes them biologically safe during the growth and application phases. In the same case, GaN-based sensor devices are smaller in size and have greater compatibility with sensing feedback. And GaN-based devices show potential for applications in a wide range of ion sensing, such as zinc ions [3], lead ions [4], ammonium ions [5], copper ions [6], cadmium ions [7], etc. However, there are fewer theoretical studies related to changes in the density of surface states and changes in the energy bands of materials due to ion adsorption. For potassium ion sensing, this can be achieved by precipitating ion-selective films in the gate region for the identification of potassium ions [8]. However, the preparation process is complicated, and the applicable environment is strict. Furthermore, potassium is one of the most important cations in the body and is involved in a number of physiological processes. If the level of potassium is too high or too low in the body, it can have an impact on health and cause functional or pathological changes in a number of systems, which is why it is particularly important to test for it. Therefore, in this paper, we simulated the change in the density of the surface states and the change in the energy bands of the material after adsorption of potassium ions with simulation software using density functional theory, and the change in the current induced by adsorption was simulated by device simulation software. We validate the direct implementation of potassium ion sensing via surface potential effects in AlGaN/GaN heterojunction HEMT devices using silicon substrates through software simulations.

## 2. Simulation and Results

### 2.1. AlGaN (001) Surface

**Figure 1:**
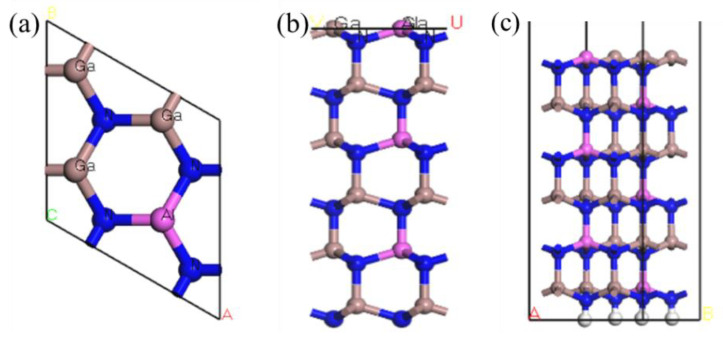
(**a**) Top view of the Al0.25Ga0.75N structure, (**b**) after the crystal structure is sliced, and (**c**) the model of the AlGaN crystal surface structure.

According to the above method, an AlGaN structure with an Al fraction of 0.25 was selected, which was obtained by replacing 25% of Ga atoms with Al. The structure of the Al atom was selected to replace the para-Ga atom, which is the most stable [9], and the surface structure was constructed after optimization to obtain the model shown in Figure 1.

### 2.2. Adsorption Energy Analysis

**Figure 2:**
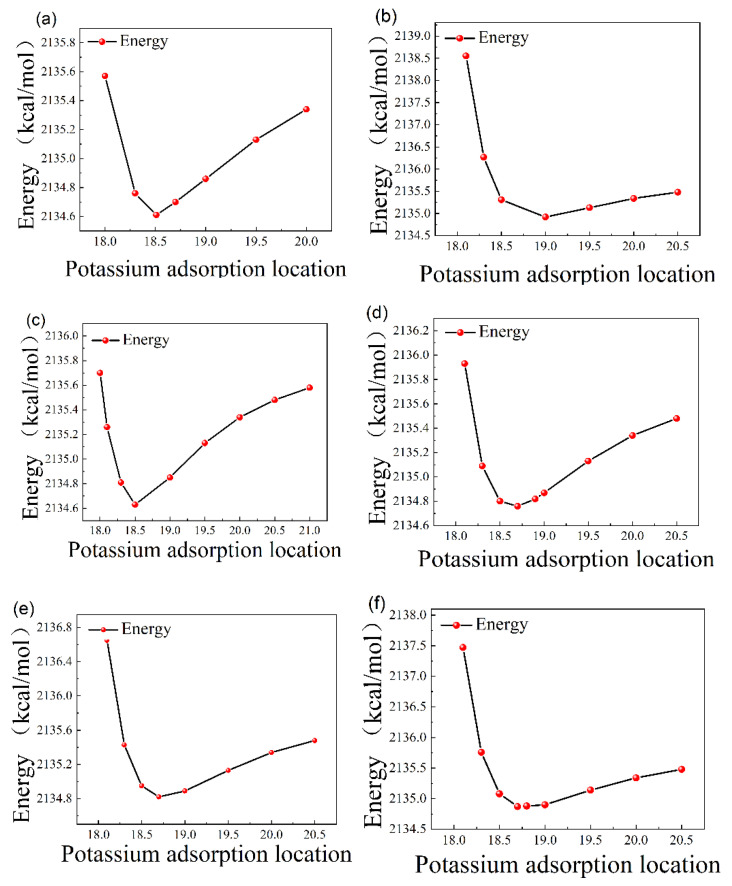
(**a**) N-top site, (**b**) Al-top site, (**c**) center site, (**d**) Ga-N bridge site, (**e**) Al-N bridge site, and (**f**) Ga-top site.

When the potassium ion was fixed above each adsorption site, it was allowed to move freely in the z-axis direction, and the curve of energy after adsorption versus the position of the potassium ion was obtained, as shown in Figure 2.

Through the above calculation of the most stable position of the six adsorption points on the AlGaN surface, the most stable adsorption position and the energy corresponding to the stable position were obtained in the six sites, the energy of the adsorbed potassium ions were calculated separately. In order to obtain the most stable adsorption structure, we calculated the adsorption energy after optimizing the structure; the calculation formula is as follows:

$$E_{ad}=\frac{1}{N_{ad}}(E_{total}-E_{sub}-N_{ad}{E_{K}}^{+})$$

where $N_{ad}$ is the number of potassium ions adsorbed, $E_{total}$ is the total energy after adsorption, $E_{sub}$ is the energy of the substrate, and ${E_{K}}^{+}$ is the energy of the potassium ions [10,11,12]; in this paper, only one potassium ion is considered to be adsorbed. According to the above formula for calculation and analysis, we can determine the adsorption energy of potassium ions in the six positions as shown in Table 1.

Adsorption energy is a measure of the strength of adsorption between an adsorbed substance and an adsorbed surface. Adsorption energy is the energy generated during the process of adsorption, in which the speed of molecular movement gradually slows down and finally stops at the surface of the adsorbed substance. So, due to the reduced speed, part of the energy is released, and this part of the energy is known as adsorption energy. The more negative the adsorption energy is, the greater the adsorption energy is and the more stable the structure is. On the contrary, the structure obtained is unstable. From the above table, regarding the adsorption energy, the energy of the potassium ions after adsorption on the AlGaN surface is lower than the energy of the substrate, indicating the increased stability of the system after adsorption. And the adsorption energy is lowest and the structure is most stable when it is above the N atom.

### 2.3. Structural Analysis

Through further analysis of the system’s structure after adsorption at each location, the table below lists the changes in the atomic layers after adsorption. $d_{ad}$ is the distance from the potassium ion to the AlGaN surface, and $d_{12}$ is the distance between the first and second layers in the surface structure. According to the calculations, there is little change in the surface structure after the adsorption of potassium ions, and no chemical bonding is created or broken.

Using Table 1 and Table 2, our analysis shows that for the N-top, Al-top and Al-N bridge sites, the distance between the two layers of atoms is the same as the value for a clean substrate, indicating that there is no change in the surface structure after the adsorption of potassium ions, but for the Al-top site, the ion-to-surface distance is the largest and the interaction between the potassium ions and the surface structure is the weakest, while for the Al-N bridge site, the adsorption energy is the largest, so compared to the other sites, the structure obtained after adsorption is unstable.

Therefore, in a comprehensive analysis, we can conclude that at the N-top site, the distance between the two layers is the same as the value when the substrate is clean, and the potassium ion is closest to the surface, with the lowest adsorption energy, at the N-top site, as the ideal adsorption site.

### 2.4. Analysis of Electrical Properties of AlGaN

**Figure 3:**
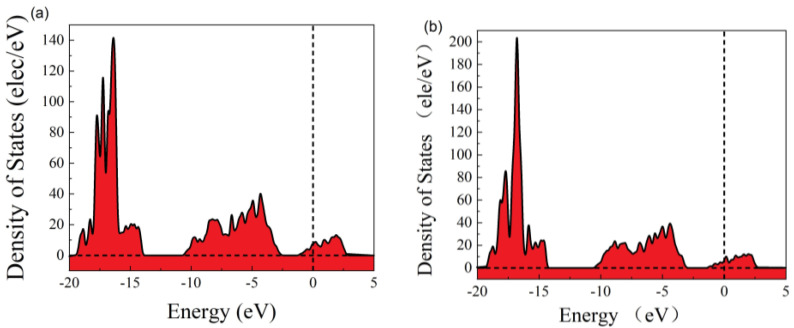
The density of states: (**a**) before adsorption and (**b**) after adsorption.

In order to better understand the change in electrical properties, we calculated the density of states before and after adsorption. It can be seen that the valence band density of states decreases, the conduction band density of states increases, the electronic states in the conduction band energy levels increase, and electrons are more easily excited into the conduction band. This shows that the band gap increases, the conduction band moves to higher energy levels, and the valence band moves to lower energy levels (Figure 3).

**Figure 4:**
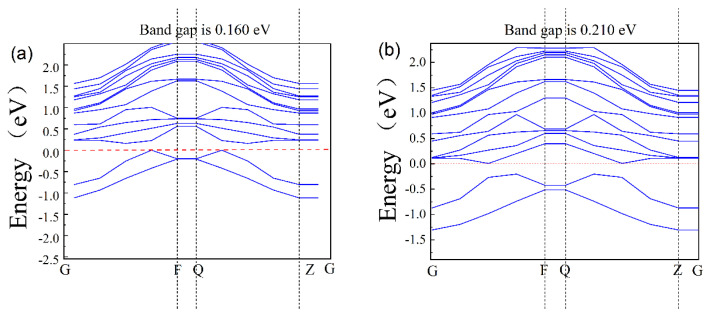
The band gap of AlGaN: (**a**) before adsorption and (**b**) after adsorption.

The band structure of the AlGaN surface is shown in Figure 4a,b, where a is the pre-adsorption and b is the post-adsorption; they are 0.160 eV and 0.210 eV, respectively. Due to the well-known DFT band gap under simulation, the calculated band value is smaller than the experimental values by 30~50%. Compared with bulk materials, the surface of AlGaN is different due to surface relations and ion adsorption, so its band structures are different. From Figure 4, we took the difference of its change, that is, adsorption of one potassium ion on the surface of AlGaN increases the value of the forbidden band width of the material by 0.05 eV.

### 2.5. HEMT Device Characterization

**Figure 5:**
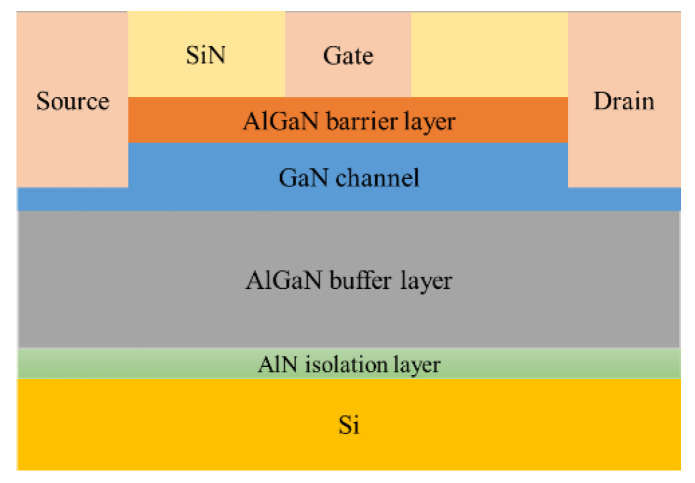
HEMT devices on silicon substrates.

**Figure 6:**
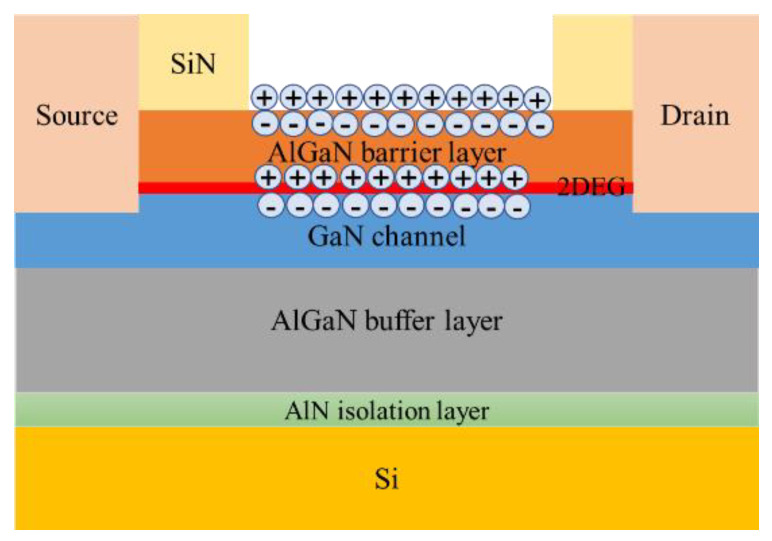
Schematic diagram of the adsorption sensing principle of the AlGaN/GaN heterostructure.

According to the parameters given in the literature to build the device model in TCAD, its structure is shown in Figure 5, and its characteristics were simulated to obtain the output characteristic curve, which is in line with the basic laws of HEMT device characteristics. Among them, the GaN sensor is called gateless in the actual preparation, as shown in Figure 6; this kind of schematic diagram can be seen in many studies related to AlGaN sensors [13,14]. The sensing process of the actual device is as follows: a surface potential on the sensing surface will form when specific ions are adsorbed onto the sensing surface, and the surface potential will cause a change in the electron concentration in the AlGaN/GaN heterostructure, thereby causing a change in the output current of the device. The surface electric potential here is equivalent to the gate voltage of the device, and it plays a role in regulating the electron concentration in the device’s channel. Therefore, in the simulation study, in order to account for the electric potential formed after the sensing area adsorbs ions, a gate needs to be added to facilitate the application of voltage in the simulation calculation.

**Figure 7:**
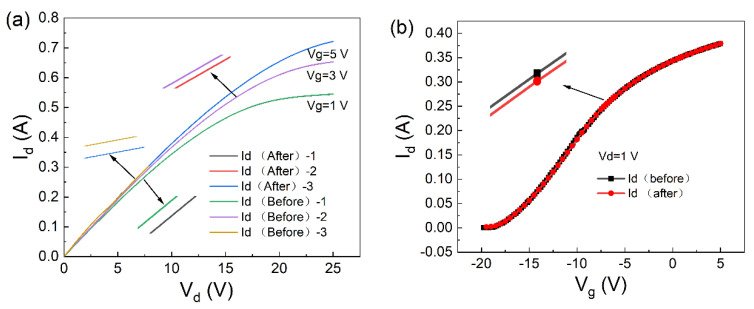
Before and after absorption of potassium ions by the AlGaN/GaN device: (**a**) output characteristic curve (Id-Vd) and (**b**) transfer characteristic curve (Id-Vg).

The parameter changes caused by the adsorption of potassium ions by the material were set using two-dimensional device simulation software. The output characteristics were obtained, Id-Vd and Id-Vg, as shown in Figure 7a,b. At a gate voltage of 0 V, the output characteristic curve of the device was calculated as shown in Figure 7. The curve shows the saturation drain currents of 544.7610 mA and 544.9399 mA, respectively. The change in the saturation of the drain current was 0.1789 mA. From the current variation curve in Figure 7, it can be seen that the trend of current change after the device adsorbs potassium ions is the same as the trend of the actual experimental data [13,14].

From the density of states analysis, it was concluded that the increase in the density of states in the conduction band and the widening of the bandgap lead to a decrease in the concentration of conduction band electrons, electron build-up occurs on the surface of AlGaN, and a positive charge is induced on the side of AlGaN close to the heterojunction, and in order to maintain the electrically neutrality at the interface, the concentration of negative charges in the channel is decreased, which leads to a decrease in the concentration of the two-dimensional electron gas. From the point of view of the electrical characteristics of the device, when the AlGaN surface absorbs some positively charged potassium ions, an equal amount of negative charge is formed on the side near the sensing surface. For the bulk of the AlGaN, in order to maintain electrical neutrality, the same amount of positive charges will be introduced near the heterostructure interface, which will produce a depletion effect on the electrons in the channel, thus reducing the concentration of 2DEG in the channel and ultimately resulting in a decrease in the output current.

Based on the above results and analysis, we can conclude that when potassium ions are adsorbed on the material surface, they can cause a change in the electrical signal, which in turn enables sensing.

## 3. Simulation Method

Since the general sensing surface will be distributed into nearly 100,000 or more atoms (different sensing area and different number of atoms), such a large number of atoms all calculated based on first principles is obviously unrealistic, so we used a few atoms to simulate the adsorption behaviors of the sensing surface, as a way to estimate or predict the device’s response to the characteristics of ion sensing. For AlGaN/GaN heterostructures, the main sensing surface is the AlGaN surface. Therefore, in this paper, the simulation is based on the AlGaN model. The DFT [15] calculation adopted in this paper uses the CASTEP module to optimize the geometry of the above model, and the generalized gradient approximation GGA-PBE is used to describe the exchange correlation energy. The cutoff energy is set to 500 eV, and the number of k-points is set to 4 × 4 × 1. Through the morphology module, the crystal growth morphology is predicted from the atomic structure, and it was determined that the AlGaN (0001) plane is the most stable during the crystal growth process, and it is the optimal orientation plane. The six-layer structure is constructed through this plane, and the vacuum layer of 15 Å is added. The dangling bonds in the bottom nitrogen layer are saturated through the addition of a pseudo-hydrogen molecule and the addition of 3/4 of the fractional charge to saturate them and immobilize the bottom three layers. Optimization is carried out using a Forcite module and finally by calculating the optimized surface structure. After the AlGaN surface model has been constructed, looking for the possible adsorption sites for potassium ions on the AlGaN (0001) surface is the next step. In order to determine the optimum adsorption site, we need to place potassium ions at different bonding sites, and the optimum adsorption site is found when the adsorption energy is at its minimum. As there are many possible adsorption sites, it is not possible to perform detailed calculations for all of them, so several symmetrical adsorption sites were selected, namely N-top, Ga-top, Al-top, center, Ga-N bridge, and Al-N bridge. We tested the equilibrium position at six positions to obtain the most stable adsorption position. By fixing the X and Y coordinates of potassium ions at the adsorption site and then changing the Z coordinates of potassium ions, the energy value of each position was recorded, the lowest energy position was found, and the equilibrium position was determined. After determining the equilibrium position, adsorption was carried out at its site to determine the change in material properties before and after adsorption. Finally, when the adsorption site was determined, the potassium ions were fixed in the site, and the adsorption caused a change in the material properties, but in practice, it was poorly measured and not intuitive. Therefore, the construction of the HEMT device model in TCAD software 2014 (Silvaco, Santa Clara, CA, USA) was based on the data provided in the literature [16], and the change value of the band gap simulated by the DFT calculations was defined for the material through custom material parameters. The change in saturated drain output current when the gate voltage is 0 V was analyzed, which then confirmed that it can be used through the surface potential effect to make AlGaN/GaN heterojunction HEMT devices for potassium ion sensing applications.

## 4. Conclusions

According to the surface potential change effect, when potassium ions are adsorbed on the AlGaN surface, a change in surface charge is induced, which alters the electrical properties of the material, resulting in an increase in the forbidden bandwidth. These changes can be sensed through the channels of AlGaN/GaN heterojunction HEMT devices and lead to changes in the output current. Therefore, the method of using the surface potential change effect can enable the sensing of potassium ions and provide a theoretical basis for the application of AlGaN/GaN heterojunction HEMT devices for potassium ion sensing.

## Figures and Tables

**Table 1:** Energy of potassium ions at different sites on the AlGaN surface.

Site	Ion	$E_{total}$ (eV)	$E_{sub}$ (eV)	${E_{K}}^{+}$ (eV)	$E_{ad}$ (eV)
N-top site	K^+^	92.5607	92.6144	14.03	−14.0837
Al-top site	K^+^	92.5698	92.6144	14.03	−14.0746
Center site	K^+^	92.5615	92.6144	14.03	−14.0829
Ga-N bridge site	K^+^	92.5672	92.6144	14.03	−14.0772
Al-N bridge site	K^+^	92.5741	92.6144	14.03	−14.0703
Ga-top site	K^+^	92.5732	92.6144	14.03	−14.0712

**Table 2:** Structural changes in the adsorption system of potassium ions at different sites.

Site	N-Top	Al-Top	Centre	Ga-N Bridge	Al-N Bridge	Ga-Top	Clean
$d_{\mathrm{ad}}$/Å	3.403	3.811	3.420	3.590	3.581	3.589	/
$d_{12}$/Å	2.4063	2.4063	2.4069	2.4068	2.4063	2.4069	2.4063

## Data Availability

Data are contained within the article.

## References

[1] Abidin M.S.Z., Hashim A.M., Sharifabad M.E., Rahman S.F.A., Sadoh T. (2011). Open-Gated pH Sensor Fabricated on an Undoped-AlGaN/GaN HEMT Structure. Sensors.

[2] Zhao L., Liu X., Gu Z., Wang J., Zhao L., Peng H., Zeng B., Zhang J., Li J. (2019). A differential extended gate-AlGaN/GaN HEMT sensor for real-time detection of ionic pollutants. Anal. Methods.

[3] Gu L., Yang S., Miao B., Gu Z., Wang J., Sun W., Wu D., Li J. (2019). Electrical detection of trace zinc ions with an extended gate-AlGaN/GaN high electron mobility sensor. Analyst.

[4] Nigam A., Bhati V.S., Bhat T.N., Dolmanan S.B., Tripathy S., Kumar M. (2019). Sensitive and Selective Detection of Pb^2+^ Ions Using 2,5-Dimercapto-1,3,4-Thiadiazole Functionalized AlGaN/GaN High Electron Mobility Transistor. IEEE Electron. Device Lett..

[5] Alifragis Y., Volosirakis A., Chaniotakis N.A., Konstantinidis G., Iliopoulos E., Georgakilas A. (2007). AlGaN/GaN high electron mobility transistor sensor sensitive to ammonium ions. Phys. Status Solidi a-Appl. Mater. Sci..

[6] Jiang X.C., Xie F., Gu Y., Dong X., Zhang X., Zhu C., Qian W., Lu N., Chen G., Yang G. (2022). L-Cysteine Functionalized Al0.18Ga0.82N/GaN High Electron Mobility Transistor Sensor for Copper Ion Detection. IEEE Trans. Electron Devices.

[7] Nigam A., Bhat T.N., Bhati V.S., Dolmanan S.B., Tripathy S., Kumar M. (2019). MPA-GSH Functionalized AlGaN/GaN High-Electron Mobility Transistor-Based Sensor for Cadmium Ion Detection. IEEE Sens. J..

[8] Alifragis Y., Volosirakis A., Chaniotakis N.A., Konstantinidis G., Adikimenakis A., Georgakilas A. (2007). Potassium selective chemically modified field effect transistors based on AlGaN/GaN two-dimensional electron gas heterostructures. Biosens. Bioelectron..

[9] Mingzhu Y., Benkang C., Guanghui H., Jing G., Honggang W., Meishan W. (2013). Theoretical study on electronic structure and optical properties of Ga0.75Al0.25N(0001) surface. Appl. Surf. Sci..

[10] Kitchin J.R. (2009). Correlations in coverage-dependent atomic adsorption energies on Pd(111). Phys. Rev. B.

[11] Bi K., Liu J., Dai Q. (2012). First-principles study of boron, carbon and nitrogen adsorption on WC(100) surface. Appl. Surf. Sci..

[12] Schimka L., Schimka L., Harl J., Stroppa A., Grüneis A., Marsman M., Mittendorfer F., Kresse G. (2010). Accurate surface and adsorption energies from many-body perturbation theory. Nat. Mater..

[13] Jia X., Chen D., Bin L., Lu H., Zhang R., Zheng Y. (2016). Highly selective and sensitive phosphate anion sensors based on AlGaN/GaN high electron mobility transistors functionalized by ion imprinted polymer. Sci. Rep..

[14] Mehandru R., Luo B., Kang B.S., Kim J., Ren F., Pearton S.J., Pan C.-C., Chen G.-T., Chyi J.-I. (2004). AlGaN/GaN HEMT based liquid sensors. Solid-State Electron..

[15] Bui K.M., Shiraishi K., Oshiyama A. (2023). Insight into the step flow growth of gallium nitride based on density functional theory. Appl. Surf. Sci..

[16] Newar T., Bhajana V.V.S.K. Modelling and comparison of Si-MOSFET and eGaN-HEMT for power converter applications using TCAD. Proceedings of the 2017 2nd IEEE International Conference on Recent Trends in Electronics, Information & Communication Technology (RTEICT).

